# Ileal microbial composition in genetically distinct chicken lines reared under normal or high ambient temperatures

**DOI:** 10.1186/s42523-022-00183-y

**Published:** 2022-04-21

**Authors:** Nima K. Emami, Lori L. Schreier, Elizabeth Greene, Travis Tabler, Sara K. Orlowski, Nicholas B. Anthony, Monika Proszkowiec-Weglarz, Sami Dridi

**Affiliations:** 1 Center of Excellence for Poultry Science, University of Arkansas, 1260 W. Maple Street, Fayetteville, AR 72701 USA; 2 United States Department of Agriculture, Agricultural Research Service, Northeast Area, Animal Biosciences and Biotechnology Laboratory, Beltsville, MD 20705 USA

**Keywords:** Heat stress, Genetic line, Microbiota, Mucosal scraping, Luminal content

## Abstract

**Background:**

Heat stress (HS) has negative effects on poultry productivity, health and welfare resulting in economic losses. Broiler chickens are particularly susceptible to HS due to their high metabolic rate and rapid growth. The commensal intestinal bacterial populations have an important physiological role in the host and could ameliorate the negative effect of HS on the host. Thus, the aim of this study was to compare changes in the ileal (IL) microbiota in four different broiler lines during HS.

**Results:**

Day-old broiler chicks from Giant Jungle Fowl (JF), Athens Canadian Random Bred (ACRB), 1995 Random Bred (L1995), and Modern Random Bred (L2015) lines were raised under thermoneutral (TN) conditions until day (d) 28. On d 29 birds were subjected to TN (24 °C) or chronic cyclic HS (8 h/d, 36 °C) condition till d 56. On d 56 two birds per pen were euthanized, and IL luminal content (IL-L) and mucosal scrapings (IL-M) were collected for bacterial DNA isolation. Libraries were constructed using V3–V4 16S rRNA primers and sequenced using MiSeq. DNA sequences were analyzed using QIIME2 platform and SILVA 132 database for alpha and beta diversity, and taxonomic composition, respectively. Functional property of microbiota was predicted using the PICRUSt 2 pipeline and illustrated with STAMP software. Shannon index was significantly elevated in IL-M under HS. β-diversity PCoA plots revealed separation of microbial community of L2015-TN from JF-TN, JF-HS, ACRB-TN, and ACRB-HS in the IL-M. PERMANOVA analysis showed a significant difference between microbial community of L1995-HS compared to ACRB-HS and JF-TN, L1995-TN compared to ACRB-HS and JF-TN, L2015-HS compared to ACRB-HS and ACRB-TN, L2015-HS compared to JF-TN, L2015-TN compared to ACRB-HS and JF-TN, and ACRB-HS compared to JF-TN in the IL-L. The impact of HS on microbial composition of IL-M was more prominent compared to IL-L with 12 and 2 taxa showing significantly different relative abundance, respectively. Furthermore, differences in microbiota due to the genetic line were more prominent in IL-M than IL-L with 18 and 8 taxa showing significantly different relative abundance, respectively. Unlike taxonomy, predicted function of microbiota was not affected by HS. Comparison of L2015 with JF or ACRB showed significant changes in predicted function of microbiota in both, IL-M and IL-L. Differences were most prominent between L2015 and JF; while there was no difference between L2015 and L1995.

**Conclusions:**

These data indicate the genetic line × temperature effect on the diversity and composition of IL microbiota. Moreover, the data showcase the effect of host genetics on the composition of IL microbiota and their predicted function. These data are of critical importance for devising nutritional strategies to maintain GIT microbial balance and alleviate the negative effects of HS on broiler chickens’ performance and health.

**Supplementary Information:**

The online version contains supplementary material available at 10.1186/s42523-022-00183-y.

## Background

Selection for high growth rate and feed efficiency over the past 80 years has made phenomenal progress in terms of breast yield, feed efficiency, and reduction of market age [1, 2] in meat-type chickens (broilers). However, modern broilers are more susceptible to environmental or bacterial challenges and cannot maintain superb performance under these challenging conditions and the negative effects of challenges on modern birds are more significant [2, 3].

Heat stress (HS) is an environmental challenge that threatens all animal and plant species [4]. Avian species, due to the unique physiology, such as higher body temperature and feathers, are more susceptible to the negative consequences of HS [5]. During the HS, core body temperature increases in broiler chickens [6], and HS negatively affects broilers performance, and GIT integrity and provokes immune responses [6, 7].

Gastrointestinal tract (GIT) microbiota has an undeniable role in performance, GIT health and development of host immune responses [8–13]. The impact of GIT microbiota on the host could be via direct host-microbe interaction or indirectly through microbial metabolites. Challenging conditions, such as HS, affects community composition and function of GIT microbiota [6, 14] and leads to the dysbiosis of GIT microbial community [8, 15]. Thus, dysbiosis of GIT microbiota during HS might contribute to poor performance, and disruption of GIT integrity.

The impact of HS on GIT microbial composition and function is not fully understood. In addition, there is no information regarding the consequences of genetic selection on broilers GIT microbiota. Therefore, we hypothesized that the susceptibility of modern broiler chickens to HS compared to their ancestors reported previously [3], might be related to their microbial community composition and function. Accordingly, the current experiment was conducted to evaluate the effect of HS and genetic selection on the ileal (IL) microbiota of broiler chickens.

## Methods

### Birds, diets, and management

The study was conducted in accordance with the recommendations in the guide for the care and use of laboratory animals of the National Institutes of Health and the protocols were approved by the University of Arkansas Animal Care and Use Committee under protocols 18,083 and 16,084. All methods were carried out in accordance with relevant guidelines and regulations. This study was performed and reported in accordance with ARRIVE guidelines (https://arriveguidelines.org/). Embryonated eggs from four lines, including Giant Jungle Fowl (JF), Athens Canadian Random Bred (ACRB), 1995 Arkansas Random Bred (L1995), and Modern Random Bred (L2015) were incubated together. Embryonated eggs were incubated in a Jamesway multi-stage incubator from embryonic d 0–18 at 37.6 °C dry bulb and 29.4 °C wet bulb. At embryonic d 18, eggs were assessed for fertility and fertile eggs were transferred by line to a single hatcher unit from embryonic d 18 to hatch (d 21) with temperature set at 36.7 °C dry bulb and 28.9 °C wet bulb. These conditions are close to ideal for the 1995 and 2015 bird, however the ACRB birds were typically incubated at a warmer temperature. Detailed characteristics of the lines were reported previously [3]. The study was conducted as a 4 × 2 factorial arrangement with 4 lines and 2 environmental conditions as main factors. Day-old broiler chicks from the four chicken lines were hatched at the University of Arkansas and vent-sexed. Males were individually wing-banded with a number and barcode, and housed in environmentally controlled chambers in the Poultry Environmental Research Laboratory (University of Arkansas). Chicks were separated by line and placed into twelve environmental chambers with each chamber consisting of two equally sized pens allowing for 24 pens total (6 pens per line). Twenty-five male chicks of the same line were randomly placed in each pen and kept at an approximate density of one bird per 0.5 m^2^ in all pens. The stocking density in this study was kept low enough for all lines at placement so that stocking density at older ages would have a minimal impact on the HS experience. All lines were allowed ample floor space, far below typical industry stocking densities at older ages. All birds had ad libitum access to commercially available standard corn-soybean meal diet and water. During the first week, birds were provided with a 23 h light/1 h dark lighting program and a 20 h light/4 h dark lighting program was used from day (d) 8–56. Rearing temperature gradually decreased from 32 °C (d 1–3) to 31 °C (d 4–6), 29 °C (d 7–10), 27 °C (d 11–14), and 24 °C from d 15 to 28. From d 29 to 42, half of the pens for each line (3 pens) were raised under TN condition; while the rest of the pens (3 per line) were subjected to chronic cyclic HS (8 h/d, 36 °C). For the chronic cyclic HS groups, from d 29 to 56, the temperature was increased to 36 °C at 9 AM and decreased to 24 °C at 5 PM.

### 16S rRNA gene sequencing and bioinformatics

On d 56, two birds per replicate were selected based on the average pen weight, euthanized by cervical dislocation, and luminal content (L) and mucosal scrapings (M) were collected from IL. Bacterial DNA was isolated using PowerSoil kit (Qiagen, Valencia, CA) and a QIAcube instrument (Qiagen) as per manufacturer’s protocol. DNA concentration was measured by NanoDrop (TermoFisher Scientific, Inc., Waltham, MA), and DNA quality was assessed by TapeStation System (Agilent Technologies, Santa Clara, CA). 16S rRNA gene amplicon libraries were generated following the 16S Metagenomic Sequencing Library Preparation workflow from Illumina (Illumina, Inc., San Diego, CA) using PCR primers targeting the variable V3–V4 region of the 16S rRNA gene. Concentration and quality of the amplicons were determined using QIAxcel DNA Hi Resolution cartridge, QIAxcel ScreenGel 1.6.0 software, and QIAxcel Advanced System (Qiagen) per manufacturing instructions. The pooled DNA library was diluted to a final concentration of 4 pM and mixed with PhiX (Illumina, Inc., 4 nmol) control (20% v/v) and pair-end 2 × 300 bp sequenced using the Illumina MiSeq platform and a MiSeq Reagent Kit v3 (Illumina, Inc). The 16S rRNA gene sequences determined in this study were deposited in the NCBI Sequence Read Archive database (SRA accession # PRJNA794190).

Quantitative Insight Into Microbial Ecology (QIIME) software package 2 (version 2021.8 or higher, http://qiime2.org) [16] was used to perform quality control and analysis of the sequence reads. Raw fastq files were demultiplexed using q2-demux and quality filtered and dereplicated with q2-dada2. Sequences with average Phred score lower than 19 were removed. Taxonomy was assigned to the amplicon sequence variants (ASVs) using the q2-feature classifier classify-sklearn naïve Bayes taxonomy classifier against the SILVA database version 132 (https://www.arb-silva.de/download/archive/qiime/). MAFFT were used for multiple ASVs sequence alignment via q2‐alignment and used to construct a phylogeny with fasttree2 via q2-phylogeny. Samples of IL-L and IL-M were rarefied to 5650 and 1335 sequences, respectively for alpha- and beta-diversity analysis. Alpha-diversity was measured using the Shannon (H), Simpson, and Chao 1 indices. Differences between alpha-diversity indices were tested using the Kruskal–Wallis test (QIIME 2). To estimate the similarity of microbial community structure between groups (beta-diversity), principal coordinate analysis (PCoA) based on Weighted UniFrac distance matrix were performed (QIIME2). To assess the association between microbial community and treatments, pairwise PERMANOVA analysis implemented in QIIME2 was performed on a Weighted UniFrac distance matrix of 48 samples. The significance of PERMANOVA was obtained by 999 permutation tests.

Relative abundance of taxa for each line and temperature were calculated at the phylum and genus levels. Relative abundance data were obtained by normalization of the phylum and genus composition to the total number of reads in each sample and were analyzed using ANOVA procedure (JMP Pro 2016) and significance between treatments were determined using LSD test (*P* < 0.05). In order to predict the function of IL-L and IL-M microbiota, data analysis was performed through the Phylogenetic Investigation of Communities by Reconstruction of Unobserved States (PICRUSt)2 pipeline [17]. Then, PICRUST output for the level 3 of the Kyoto Encyclopedia of Genes and Genomes (KEGG) [18] were analyzed and illustrated with Statistical Analysis of Metagenomic Profiles (STAMP) software version 2.1.3 [19]. Within STAMP analysis, ANOVA followed by two group comparison was performed using Welsh t-test with Benjamini–Hochber false discovery rate (FDR) analysis [20].

## Results

### Ileal luminal content

**Fig. 1:**
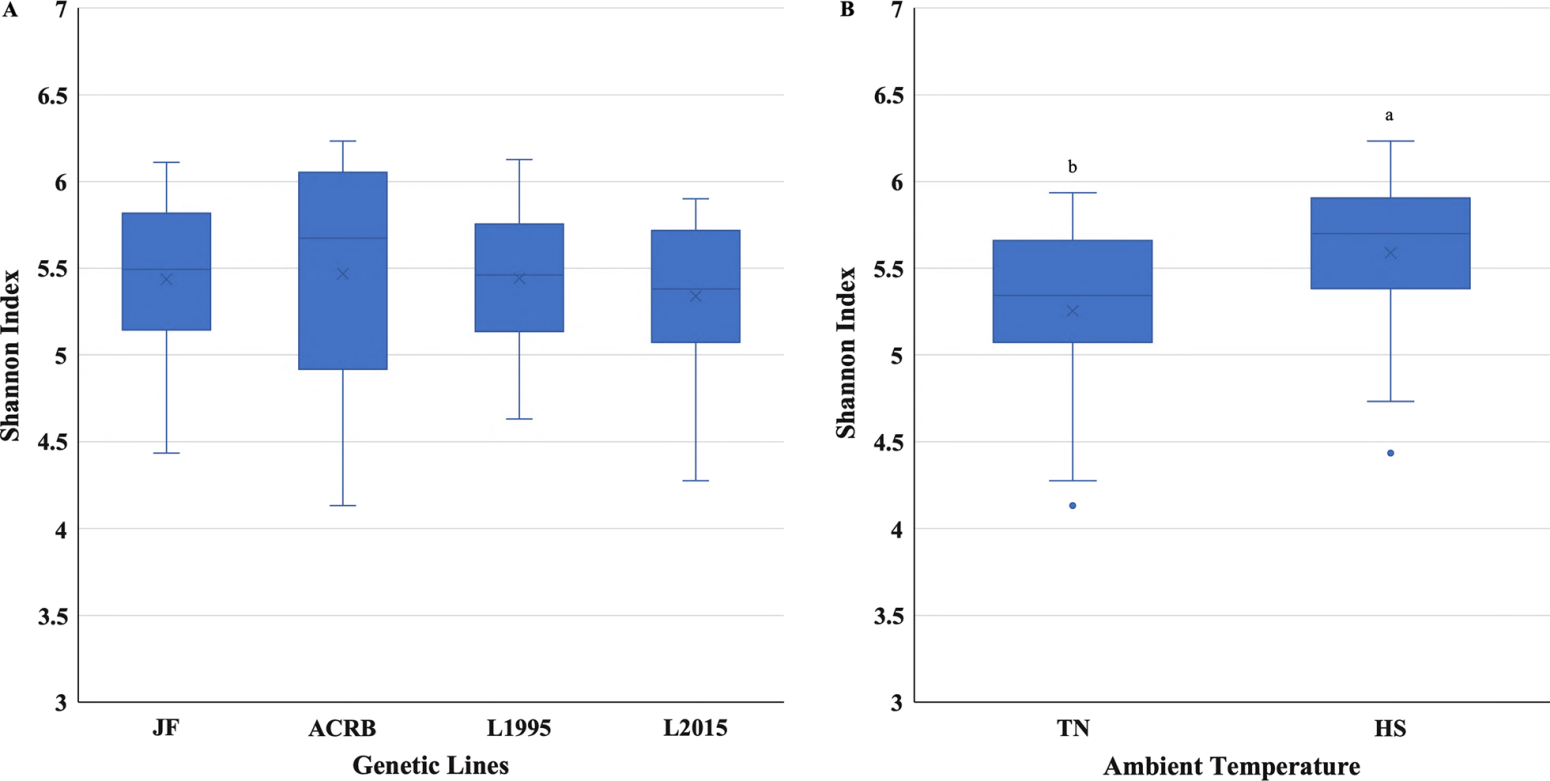
Alpha diversity in the ileal content of Jungle Fowl (JF), Athens Canadian Random Bred (ACRB), 1995 Random Bred (L1995), and 2015 Modern Random Bred (L2015) chicken lines raised under thermoneutral (TN) or heat stress (HS) condition (8 h/d; 36 °C) on d 56. **A** Shanon’s diversity index for genetic lines. **B** Shanon’s diversity index for ambient temperature. Boxplots show the quartiles, median, and extremities of the values

**Fig. 2:**
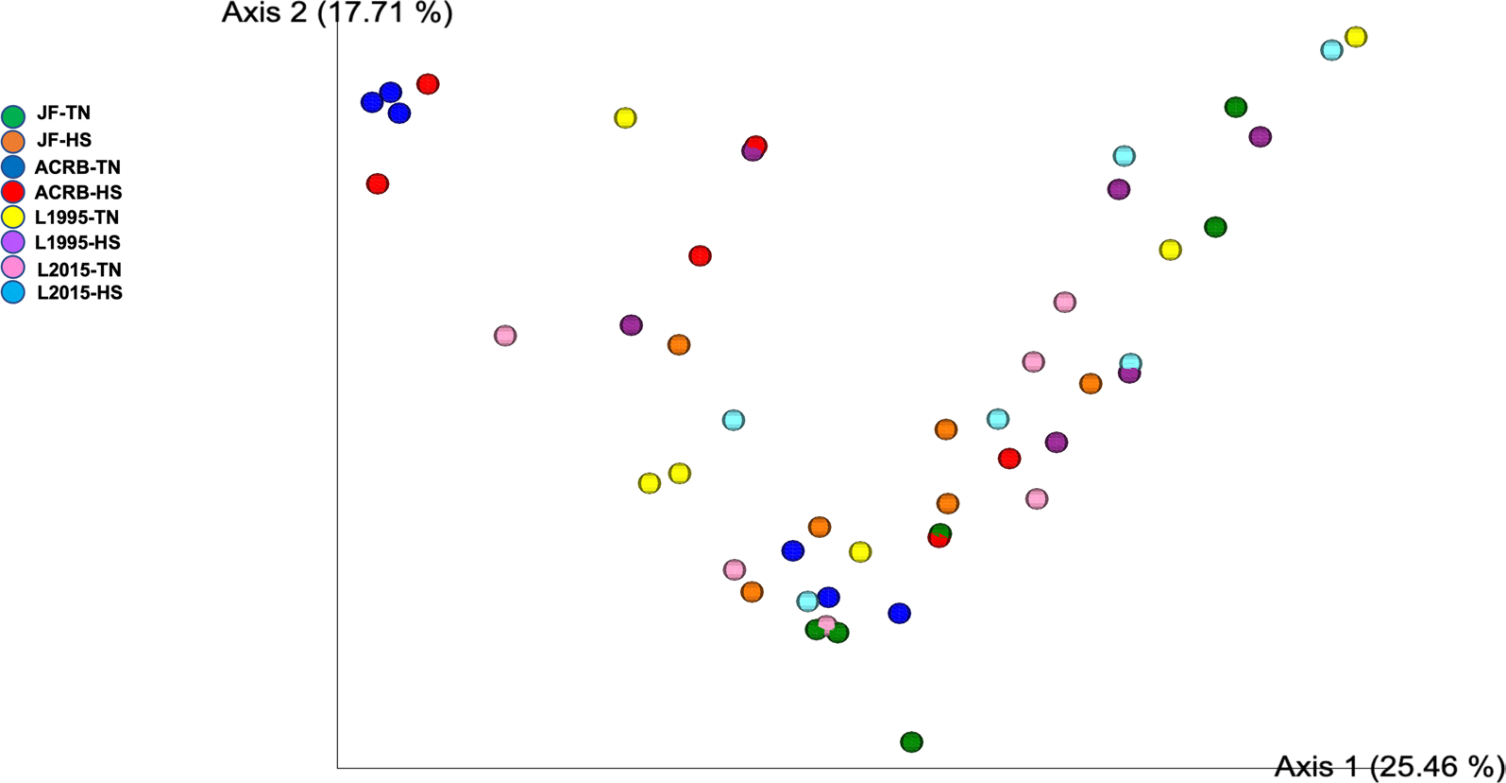
Beta diversity in the ileal content of Jungle Fowl (JF), Athens Canadian Random Bred (ACRB), 1995 Random Bred (L1995), and 2015 Modern Random Bred (L2015) chicken lines raised under thermoneutral (TN) or heat stress (HS) condition (8 h/d; 36 °C) on d 56. 2D principal coordinate analysis (PCoA) plot is based on weighted UniFrac distance matrix. Each sphere represents a sample

Alpha-diversity indices are shown in Fig. 1, and Additional file 1: Table S1. Shannon and Chao 1 indices showed that there was no significant interaction between temperature and genetic line on richness or evenness of IL-L microbial community. There were no significant differences in bacterial diversity (Shannon and Chao 1 indices) among genetic lines (Fig. 1A, and Additional file 1: Table S1), while HS birds had higher Shannon and Chao 1 indices in comparison to TN birds (Fig. 1B, and Additional file 1: Table S1). Simpson index showed that neither was there a significant interaction between temperature and genetic line, nor was there a significant effect of the main factors on richness or evenness of IL-L microbial community (data not shown). β-diversity PCoA plots revealed no separation of microbial communities due to genetic line × temperature interaction (Fig. 2). PERMANOVA analysis showed a significant (*P* = 0.001) genetic line × temperature interaction (Additional file 1: Table S2). There was a significant difference in β-diversity between microbial community of L1995-HS compared to ACRB-HS and JF-TN, L1995-TN compared to ACRB-HS and JF-TN, L2015-HS compared to ACRB-HS and ACRB-TN, L2015-HS compared to JF-TN, L2015-TN compared to ACRB-HS and JF-TN, and ACRB-HS compared to JF-TN.

**Fig. 3:**
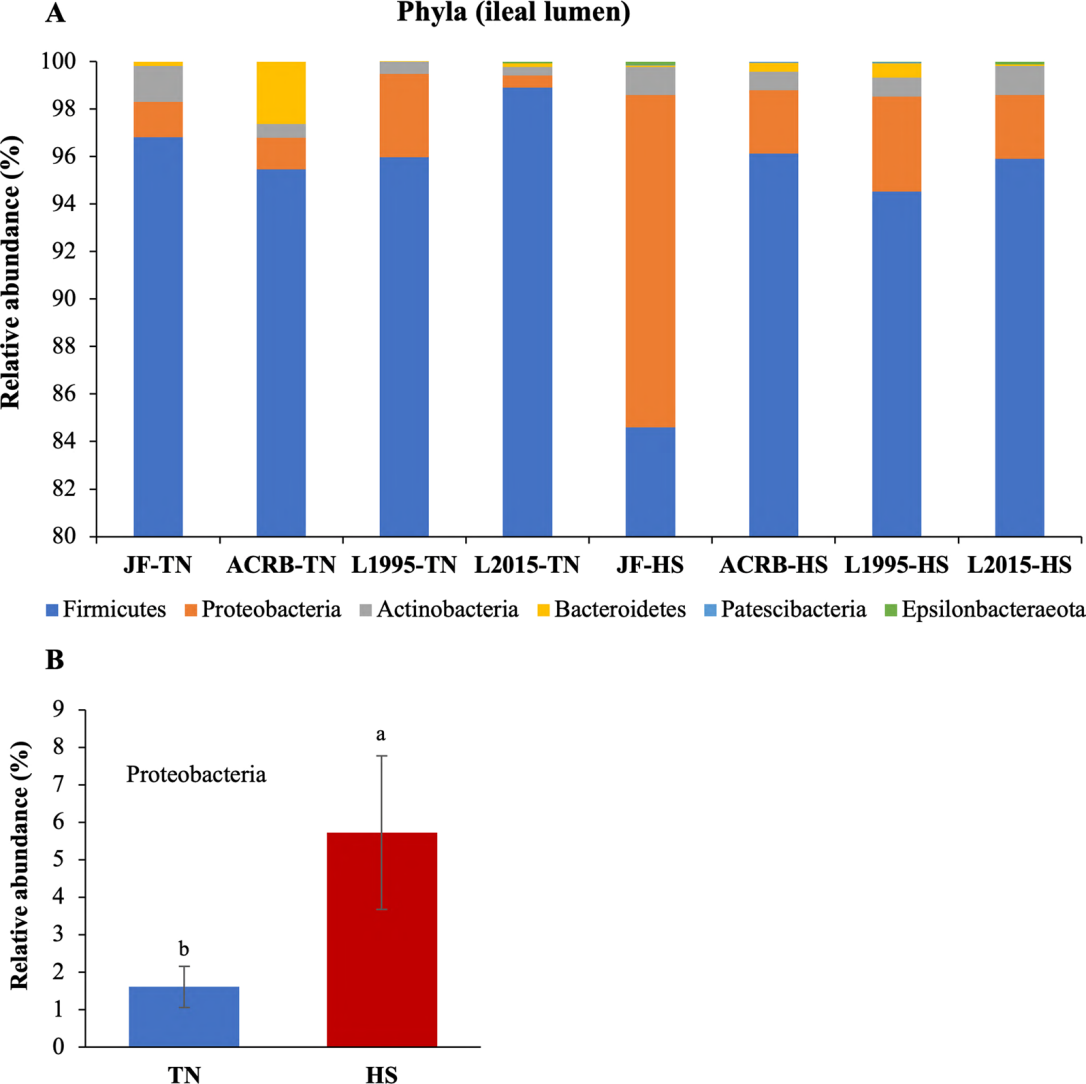
Changes in relative bacterial abundance (%) in the ileal content of Jungle Fowl (JF), Athens Canadian Random Bred (ACRB), 1995 Random Bred (L1995), and 2015 Modern Random Bred (L2015) chicken lines raised under thermoneutral (TN) or heat stress (HS) condition (8 h/d; 36 °C) on d 56 at phylum level. **A** Taxonomic profile of ileal content microbiota. **B** Effect of ambient temperature on the relative abundance of Proteobacteria. Different letters denote statistically significant (*P* < 0.05) differences

**Fig. 4:**
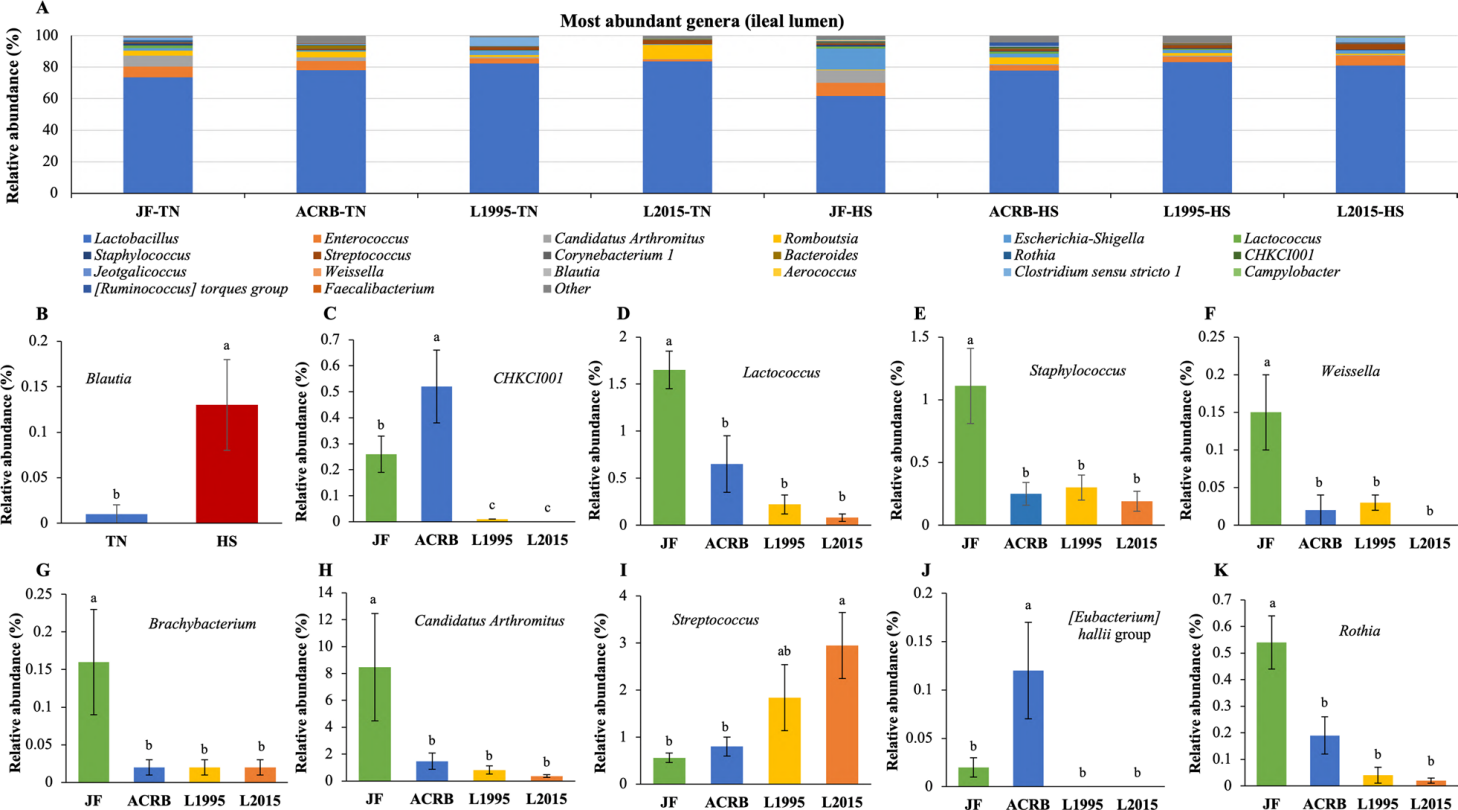
Changes in relative bacterial abundance (%) in the ileal content of Jungle Fowl (JF), Athens Canadian Random Bred (ACRB), 1995 Random Bred (L1995), and 2015 Modern Random Bred (L2015) chicken lines raised under thermoneutral (TN) or heat stress (HS) condition (8 h/d; 36 °C) on d 56 at genus level. **A** Taxonomic profile (top 20 genera) of ileal content microbiota. **B** Effect of ambient temperature on the relative abundance of *Blautia*. Effect of genetic line on relative abundance of **C** *CHKCI001*, **D** *Lactococcus*, **E** *Staphylococcus*, **F** *Weissella*, **G** *Brachybacterium*, **H** *Candidatus Arthromitus*, **I** *Streptococcus*, **J** *[Eubacterium] hallii* group, and **K** *Rothia*. Different letters denote statistically significant (*P* < 0.05) differences

The relative abundance of taxa at the phylum level is shown in Fig. 3A with Firmicutes, Proteobacteria, and Actinobacteria as the top 3 phyla. There was no effect of genetic line × temperature interaction on relative abundance of taxa at the phylum level. However, relative abundance of Proteobacteria significantly increased due to the HS (Fig. 3B). Changes in relative abundance of the top 20 genera are shown in Fig. 4A with *Lactobacillus*, *Enterococcus*, and *Candidatus Arthromitus* as the top 3 genera. There was no effect of genetic line × temperature on the relative abundance of genera. However, the relative abundance was affected by genetic line and HS (Fig. 4B–K). Relative abundance of *Blautia* was significantly increased due to HS (Fig. 4B).

Relative abundance of *Lactococcus* (Fig. 4D), *Staphylococcus* (Fig. 4E), *Weissella* (Fig. 4F), *Brachybacterium* (Fig. 4G), *Candidatus Arthromitus* (Fig. 4H), and *Rothia* (Fig. 4K) were significantly higher in JF compared to other genetic lines. Relative abundance of *CHKCI001* (Fig. 4C), and *[Eubacterium] hallii* group (Fig. 4J) were significantly higher in ACRB birds compared to other groups. Finally, relative abundance of *Streptococcus* was significantly higher in L2015 compared to JF and ACRB (Fig. 4I).

**Fig. 5:**
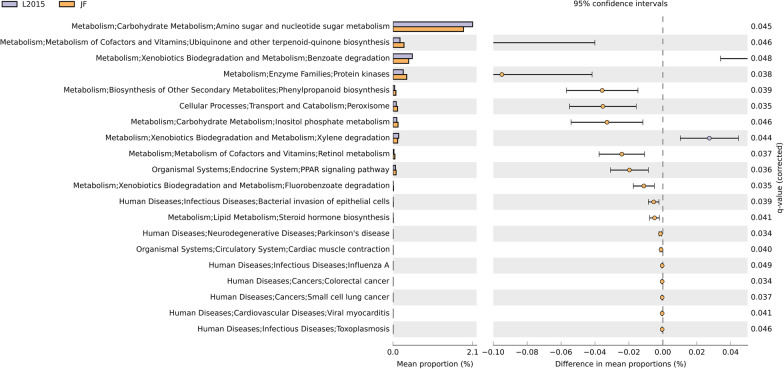
Predicted functions of ileal content microbiota in modern random bred chickens (L2015) compared to their ancestor jungle fowl (JF) on d 56. Differentially regulated metabolic pathways are shown. (n = 12/genetic line)

**Fig. 6:**
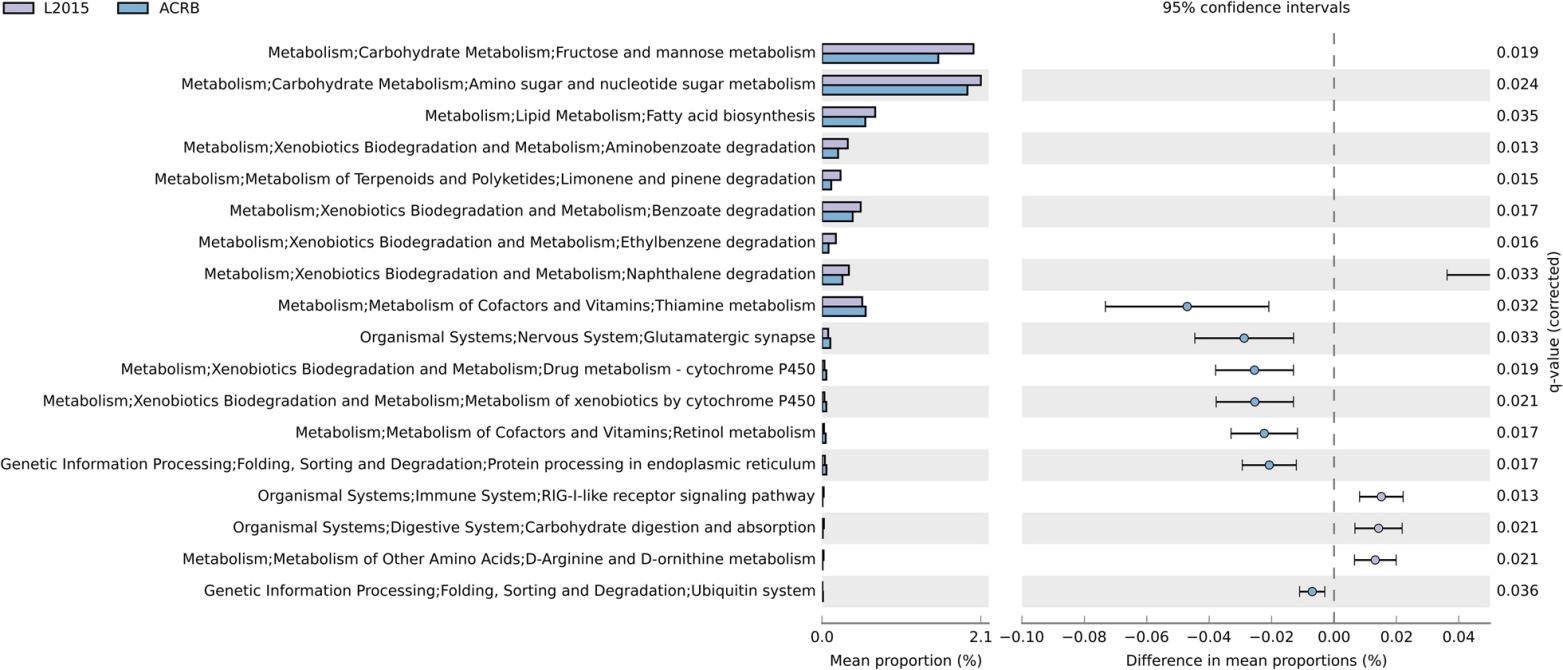
Predicted functions of ileal content microbiota in modern random bred chickens (L2015) compared to Athens-Canadian Random bred chickens (ACRB) on d 56. Differentially regulated metabolic pathways are shown. (n = 12/genetic line)

The predicted function of the microbiota is shown in Figs. 5 and 6. There was no difference in predicted function of microbiota among TN and HS (data not shown). Furthermore, there was no difference in predicted function of microbiota among L2015 and L1995 (data not shown). There were 20 pathways at KEGG level 3 with distinctive enrichment between L2015 and JF (Fig. 5). There were 18 pathways at KEGG level 3 with distinctive enrichment between L2015 and ACRB (Fig. 6). Among these, the L2015 microbiota had higher numbers of functional genes involved in carbohydrate metabolism including amino sugar and nucleotide sugar metabolism, and inositol phosphate metabolism compared to JF. Compared to ACRB, genes involved in carbohydrate metabolism including amino sugar and nucleotide sugar metabolism, and fructose and mannose metabolism were enriched in L2015. Functional genes involved in xenobiotics biodegradation and metabolism were enriched in L2015 compared to both JF and ACRB. This included higher benzoate degradation, and xylene degradation in L2015 compared to JF, and higher aminobenzoate degradation, limonene and pinene degradation, benzoate degradation, ethylbenzene degradation, and naphthalene degradation compared to ACRB. Interestingly, compared to L2015, functional genes involved in metabolism of cofactors and vitamins were enriched in both JF and ACRB. In comparison with L2015, JF had more genes involved in ubiquinone and other terpenoid-quinone biosynthesis, and retinol metabolism; while, ACRB had higher genes involved in retinol metabolism and thiamine metabolism.

### Ileal mucosal scrapings

**Fig. 7:**
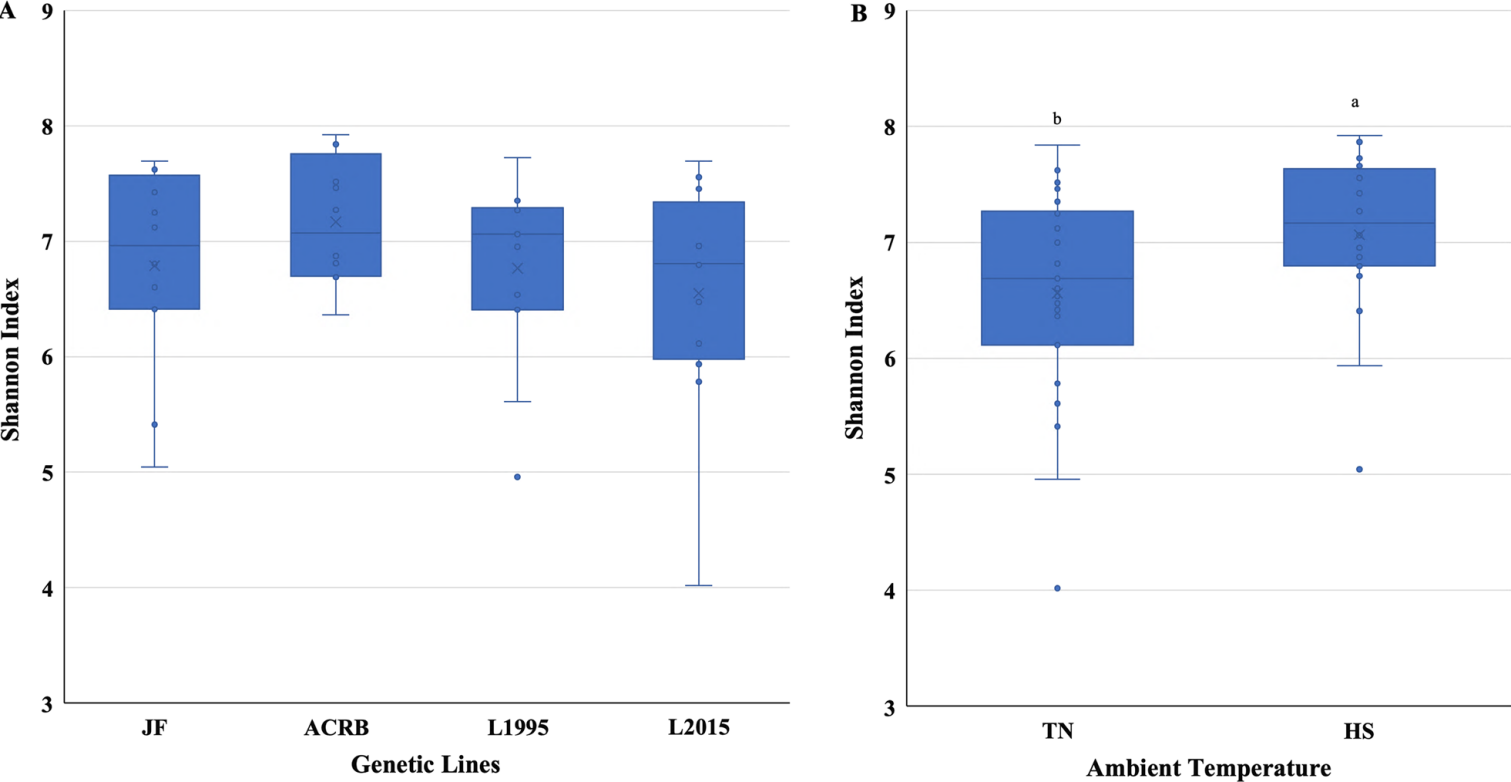
Alpha diversity in the ileal mucosa of Jungle Fowl (JF), Athens Canadian Random Bred (ACRB), 1995 Random Bred (1995), and 2015 Modern Random Bred (2015) chicken lines raised under thermoneutral (TN) or heat stress (HS) condition on d 56. Shanon’s diversity index for genetic lines (**A**). Shanon’s diversity index for ambient temperature (**B**). Boxplots show the quartiles, median, and extremities of the values

**Fig. 8:**
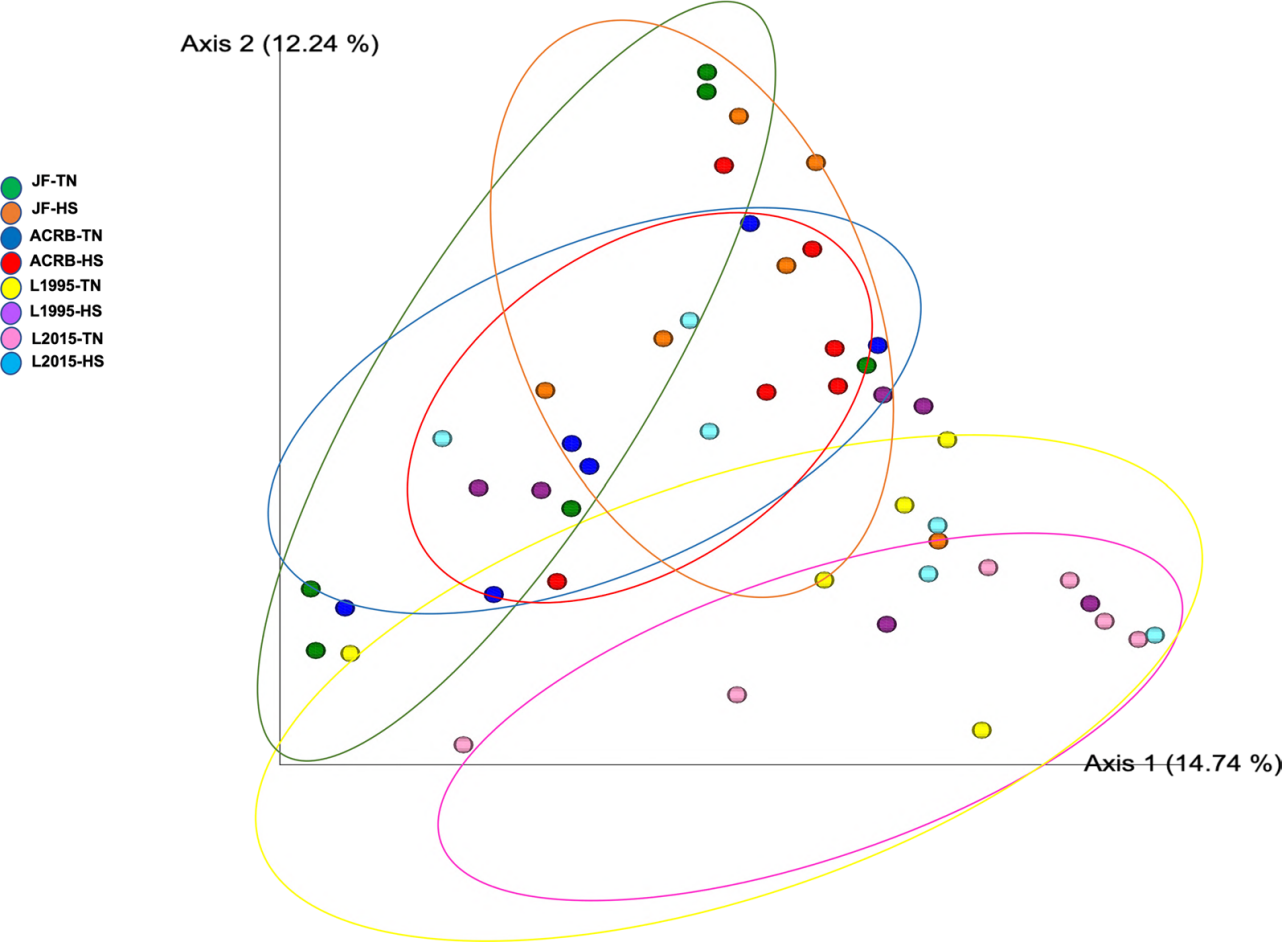
Beta diversity in the ileal mucosa of Jungle Fowl (JF), Athens Canadian Random Bred (ACRB), 1995 Random Bred (1995), and 2015 Modern Random Bred (2015) chicken lines raised under thermoneutral (TN) or heat stress (HS) condition on d 56. 2D principal coordinate analysis (PCoA) plot is based on weighted UniFrac distance matrix. Each sphere represents a sample

Alpha-diversity indices are shown in Fig. 7, and Additional file 1: Table S3. Shannon and Simpson indices showed that there was no significant interaction between temperature and genetic line on richness or evenness of IL-L microbial community. There were no significant differences in bacterial diversity (Shannon and Simpson indices) among genetic lines (Fig. 7A, and Additional file 1: Table S3), while HS birds had higher Shannon and Simpson indices in comparison to TN birds (Fig. 7B, and Additional file 1: Table S3). Chao 1 index showed that neither was there a significant interaction between temperature and genetic line, nor was there a significant effect of the main factors on richness or evenness of IL-L microbial community (data not shown). β-diversity PCoA plots revealed separation of microbial communities due to genetic line × temperature interaction (Fig. 8). Microbial community of L2015-TN was separated from JF-TN, JF-HS, ACRB-TN, and ACRB-HS (Fig. 8). PERMANOVA analysis showed a significant (*P* = 0.041) genetic line × temperature interaction (Additional file 1: Table S4). Diversity of microbial community was significantly different between JF-HS compared to ACRB-TN.

**Fig. 9:**
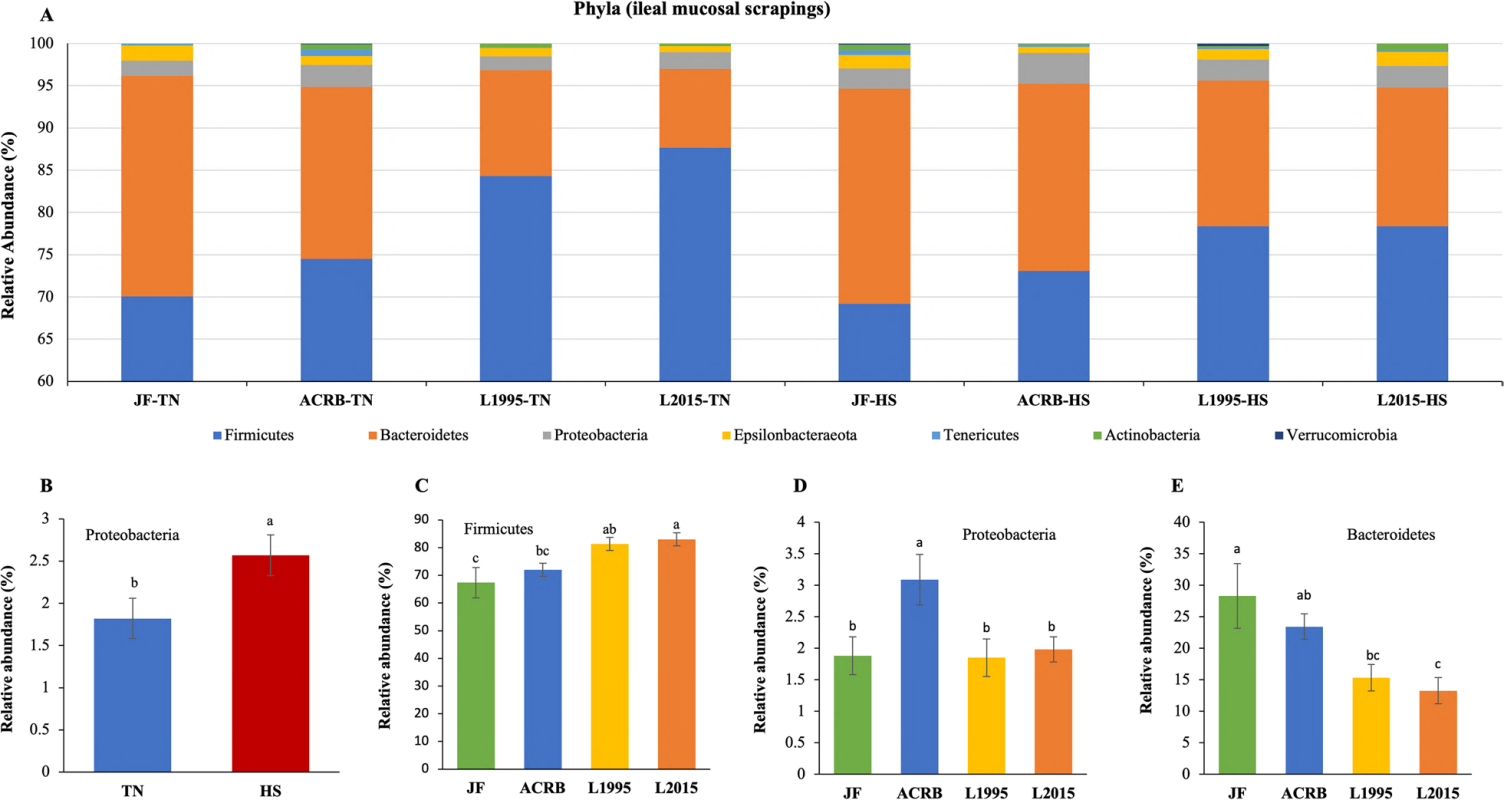
Changes in relative bacterial abundance (%) in the ileal mucosal scrapings of Jungle Fowl (JF), Athens Canadian Random Bred (ACRB), 1995 Random Bred (L1995), and 2015 Modern Random Bred (L2015) chicken lines raised under thermoneutral (TN) or heat stress (HS) condition (8 h/d; 36 °C) on d 56 at phylum level. **A** Taxonomic profile of ileal content microbiota. **B** Effect of ambient temperature on the relative abundance of Proteobacteria. Effect of genetic line on relative abundance of **C** Firmicutes, **D** Proteobacteria, and **E** Bacteroidetes. Different letters denote statistically significant (*P* < 0.05) differences

**Fig. 10:**
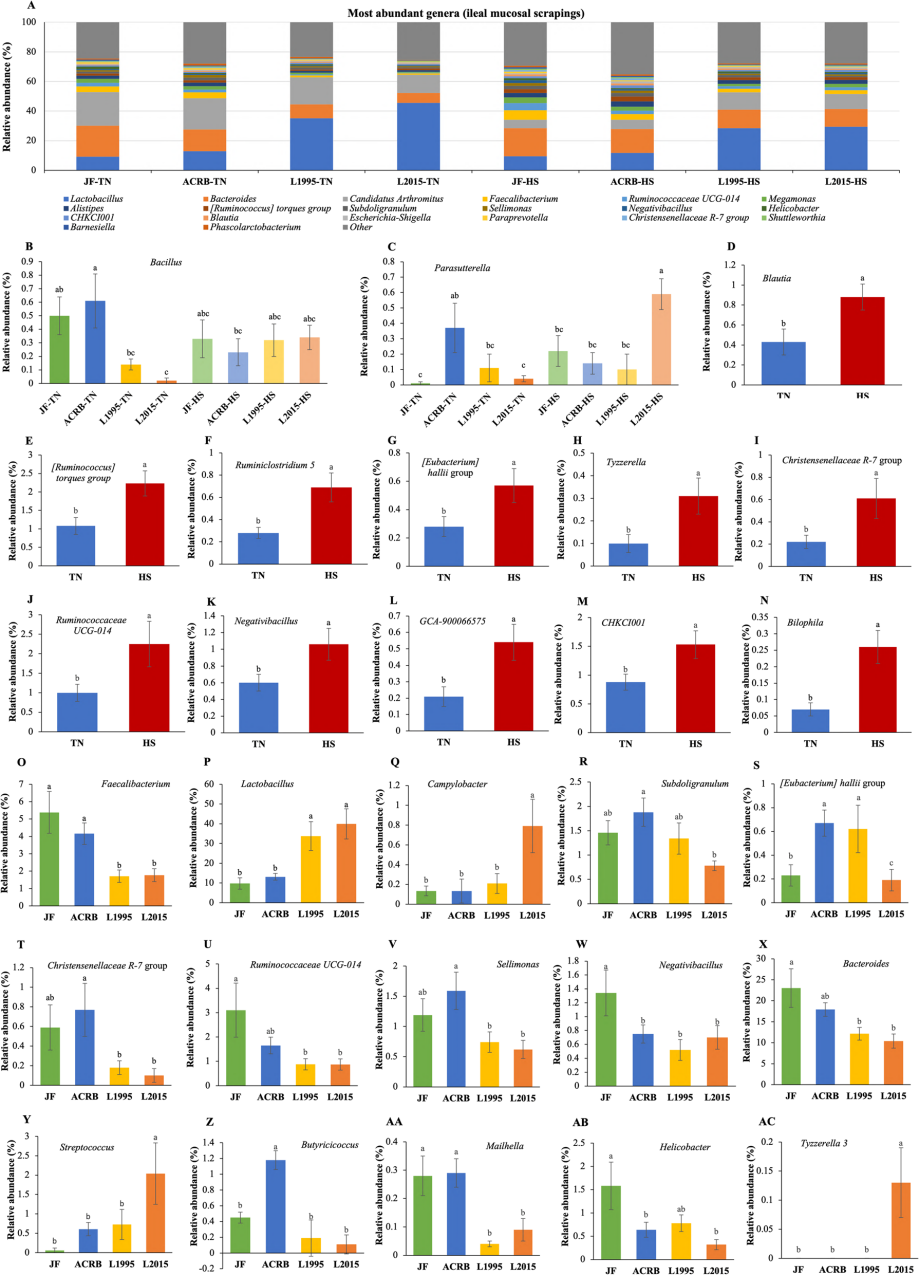
Changes in relative bacterial abundance (%) in the ileal mucosal scrapings of Jungle Fowl (JF), Athens Canadian Random Bred (ACRB), 1995 Random Bred (L1995), and 2015 Modern Random Bred (L2015) chicken lines raised under thermoneutral (TN) or heat stress (HS) condition (8 h/d; 36 °C) on d 56 at genus level. **A** Taxonomic profile (top 20 genera) of ileal mucosal scrapings microbiota. Genetic line × temperature interaction effect on **B** *Bacillus,* and **C** *Parasutterella*. Effect of ambient temperature on the relative abundance of **D** *Blautia*, **E** *[Ruminococcus] torques* group, **F** *Ruminiclostridium 5*, **G** *[Eubacterium] hallii* group, **H** *Tyzzerella*, **I** *Christensenellaceae R-7 group*, **J** *Ruminococcaceae UCG-014*, **K** *Negativibacillus*, **L** *GCA-900066575*, **M** *CHKCI001*, and **N** *Bilophila*. Effect of genetic line on relative abundance of **O** *Faecalibacterium*, **P** *Lactobacillus*, **Q** *Campylobacter*, **R** *Subdoligranulum*, **S** *[Eubacterium] hallii group*, **T** *Christensenellaceae R-7* group, **U** *Ruminococcaceae UCG-014*, **V** *Sellimonas*, **W** *Negativibacillus*, **X** *Bacteroides*, **Y** *Streptococcus*, **Z** *Butyriciococcus*, **AA** *Mailhella*, **AB** *Helicobacter*, and **AC** *Tyzzerella 3*. Different letters denote statistically significant (*P* < 0.05) differences

The relative abundance of taxa at the phylum level is shown in Fig. 9A with Firmicutes, Bacteroidetes, and Proteobacteria as the top 3 phyla. There was no effect of genetic line × temperature interaction on relative abundance of taxa at the phylum level. However, relative abundance of Proteobacteria significantly increased due to the HS (Fig. 9B). In addition, L2015 had significantly higher relative abundance of Firmicutes (Fig. 9C) and lower relative abundance of Bacteroidetes (Fig. 9E) compared to both JF and ACRB. Finally, relative abundance of Proteobacteria was significantly higher in ACRB compared to other genetic lines (Fig. 9D). Changes in relative abundance of the top 20 genera are shown in Fig. 10A with *Lactobacillus*, *Bacteroides*, and *Candidatus Arthromitus* as the top 3 genera. The genetic line × temperature interaction effect was significant on relative abundance of *Bacillus* and *Parasutterella* (Fig. 10B, C). Under TN conditions, relative abundance of *Bacillus* was significantly lower in L1995 and L2015 compared to ACRB, while there was no difference among genetic lines under HS (Fig. 10B). Relative abundance of *Parasutterella* was significantly higher in ACRB-TN compared to JF-TN and L2015-TN, while under HS, relative abundance of *Parasutterella* was significantly higher in L2015 compared to other genetic lines (Fig. 10C). Relative abundance of 11 genera including *Blautia* (Fig. 10D), *[Ruminococcus] torques* group (Fig. 10E), *Ruminiclostridium 5* (Fig. 10F), *[Eubacterium] hallii* group (Fig. 10G), *Tyzzerella* (Fig. 10H), *Christensenellaceae R-7* group (Fig. 10I), *Ruminococcaceae UCG-014* (Fig. 10J), *Negativibacillus* (Fig. 10K), *GCA-900066575* (Fig. 10L), *CHKCI001* (Fig. 10M), and *Bilophila* (Fig. 10N) were increased due to HS. There were 15 genera with differential relative abundance among the genetic lines (Fig. 10O–AC). Relative abundance of *Lactobacillus* was significantly higher in L1995 and L2015 compared to JF and ACRB (Fig. 10P). Besides, relative abundance of *Faecalibacterium* (Fig. 10O) and *Mailhella* (Fig. 10AA) were significantly lower in L1995 and L2015 compared to JF and ACRB. Furthermore, relative abundance of *Campylobacter* (Fig. 10Q), *Streptococcus* (Fig. 10Y), and *Tyzerella 3* (Fig. 10AC) were significantly higher in L2015 compared to other lines. JF had significantly higher relative abundance of *Negativibacillus* compared to other genetic lines (Fig. 10W). In addition, relative abundance of *Ruminococcoceae UCG-014* (Fig. 10U), and *Bacteroides* (Fig. 10X) were significantly higher in JF compared to L1995 and L2015. Finally, relative abundance of *Butyriciococcus* was significantly higher in ACRB compared to other genetic lines (Fig. 10Z).

**Fig. 11:**
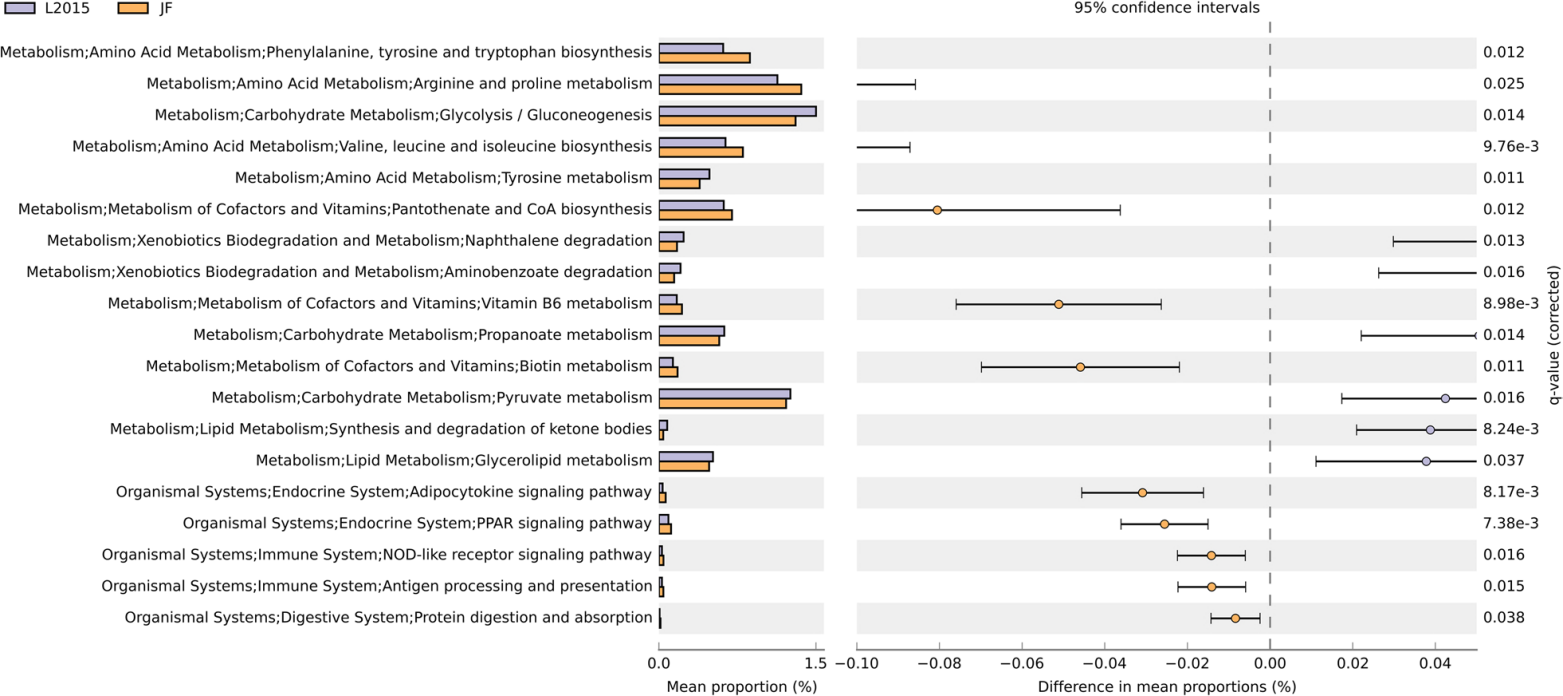
Predicted functions of ileal mucosa microbiota in modern random bred chickens (L2015) compared to their ancestor jungle fowl (JF) on d 56. Differentially regulated (top 20) metabolic pathways are shown. (n = 12/genetic line)

**Fig. 12:**
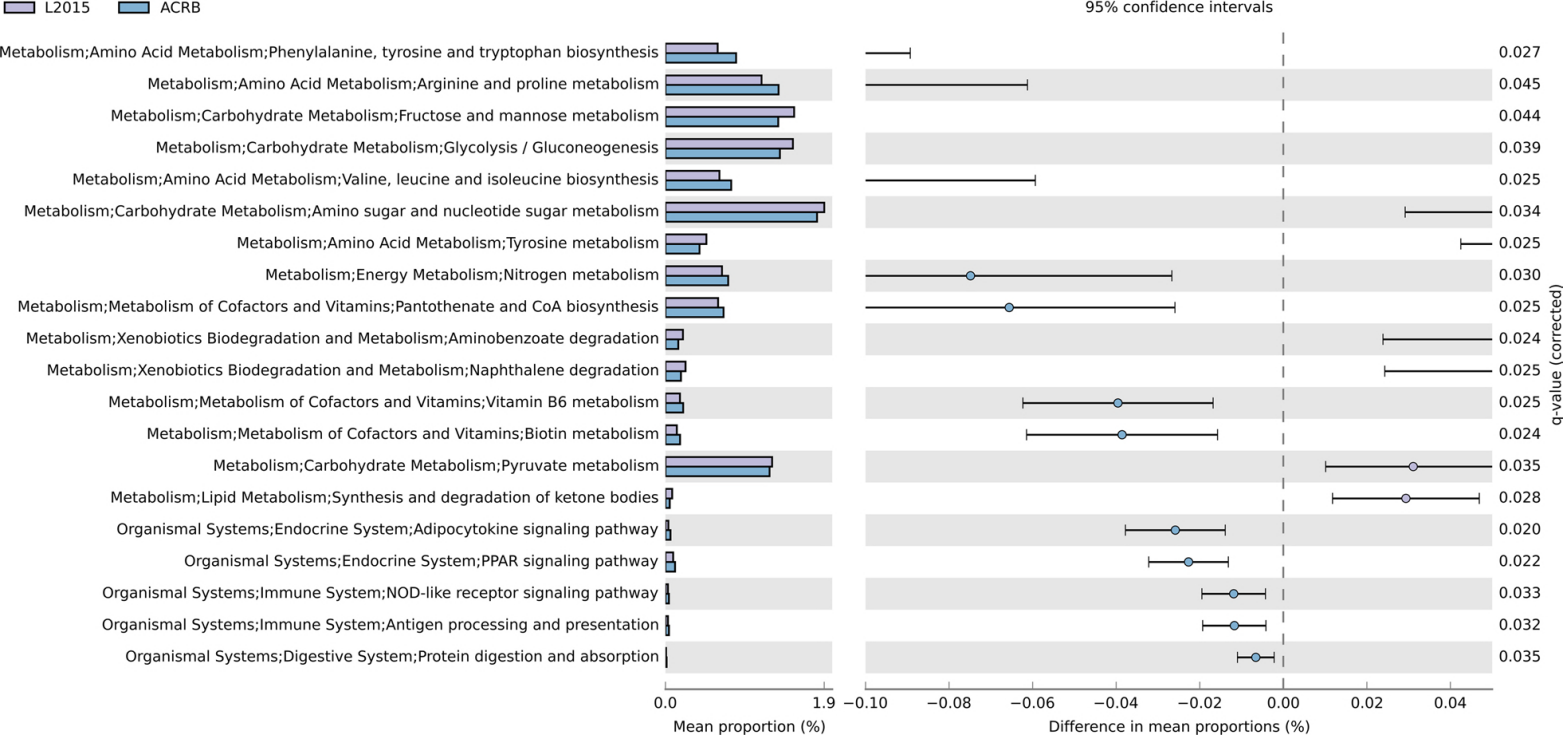
Predicted functions of ileal mucosa microbiota in modern random bred chickens (L2015) compared to Athens-Canadian Random bred chickens (ACRB) on d 56. Differentially regulated (top 20) metabolic pathways are shown. (n = 12/genetic line)

Predicted Function of the microbiota is shown in Figs. 11 and 12. There was no difference in predicted function of microbiota among TN and HS (data not shown). Furthermore, there was no difference in predicted function of microbiota among L2015 and L1995 (data not shown). There were 76 pathways at KEGG level 3 with distinctive enrichment between L2015 and JF (Fig. 11 and Additional file 1: Fig. S1). There were 58 pathways at KEGG level 3 with distinctive enrichment between L2015 and ACRB (Fig. 12 and Additional file 1: Fig. S2). Among these, the L2015 microbiota had higher numbers of functional genes involved in carbohydrate metabolism including glycolysis/gluconeogenesis, pyruvate metabolism, and propanoate metabolism compared to JF. Compared to ACRB, the L2015 microbiota had higher numbers of functional genes involved in carbohydrate metabolism including glycolysis/gluconeogenesis, pyruvate metabolism, fructose and mannose metabolism, and amino sugar and nucleotide sugar metabolism. Furthermore, functional genes involved in lipid metabolism including synthesis and degradation of ketone bodies, and glycerolipid metabolism were enriched in L2015 compared to both JF and ACRB. Finally, genes involved in xenobiotics biodegradation and metabolism were enriched in L2015 compared to both JF and ACRB.

Compared to L2015, functional genes involved in immune system including NOD-like receptor signaling pathway, and antigen processing and presentation were enriched in both JF and ACRB. Furthermore, functional genes involved in the endocrine system including adipocytokine signaling pathway, and PPAR signaling pathway were enriched in both JF and ACRB in comparison to L2015. Finally, compared to L2015, functional genes involved in amino acid metabolism and metabolism of cofactors and vitamins including arginine and proline metabolism, valine, leucine and isoleucine biosynthesis, pantothenate and CoA biosynthesis, vitamin B6 metabolism, and biotin metabolism were enriched in both JF and ACRB.

## Discussion

Understanding the interplay between host and its microbiota is of great importance especially regarding their dynamic in various environmental conditions. To our knowledge, this is the first experiment comparing the genetic line × temperature interaction effect on GIT microbiota in poultry. The effect of high ambient temperature on the alpha-diversity of GIT microbiota depends on the duration and intensity of heat exposure, and GIT segment [21]. In our study, alpha diversity was significantly increased in IL-L and IL-M due to HS, indicating a shift in the GIT microbial community which might be due to the favorable environment for the overgrowth of opportunistic bacteria and partially justify the negative effects of HS on performance and health in L1995 and L2015 as previously reported by our group [3]. Previously, Wang et al. [22] reported higher alpha diversity indices in the ileal content of chickens raised under high ambient temperature compared to TN condition [22]. In this experiment, we indicated separation of microbial community of L2015-TN from JF-TN, JF-HS, ACRB-TN, and ACRB-HS in the IL-M. Similarity of microbial community of L2015-HS with JF and ACRB might be due to reduction in feed intake due to HS and its impact on the diversity of microbial community. Genetic lines such as JF and ACRB have lower feed intake compared to modern commercial strains [3] and feed intake significantly decrease during HS in L2015 and L1995, but not in JF and ACRB [3].

Composition and function of microbial population in various segments of the GIT can influence feed efficiency, although the extent of interplay between the host and microbiome is unclear [23]. Reduction in relative abundance of *Bacillus* under HS in the IL-M of ACRB, but not other genetic lines could be indicative of higher reliance of this genetic line on *Bacillus* species for proper physiological function. Previous reports show the alleviating effects of *Bacillus*-based probiotics during HS [24], thus the effect of supplementation of *Bacillus*-based probiotics on the performance of ACRB under HS needs further investigation. Relative abundance of *Parasutterella* was significantly higher in ACRB-TN compared to JF-TN and L2015-TN, while under HS relative abundance of *Parasutterella* was significantly higher in L2015 compared to other genetic lines. *Parasutterella* is a strict anaerobe belonging to the phylum Proteobacteria [25] and positive correlation between relative abundance of *Parasutterella* and performance was previously reported in chickens [26], which is not the case in our experiment. We observed an increase in the relative abundance of Proteobacteria in IL-L and ILM due to the HS. Performance data from this trial were previously published [3] indicating an association between an increase in abundance of Proteobacteria and reduction in feed intake. Furthermore, we indicated higher relative abundance of *Blautia* in IL-L and IL-M, and higher relative abundance of *Ruminiclostridium 5* in IL-M of HS compared to TN group. *Ruminiclostridium 5* and *Blautia* are correlated with higher abdominal fat percentage in chickens [27], and higher fat pad is one of the results of HS in broiler chickens [6]. Furthermore, higher abundance of some species of *Blautia* were reported in chickens with high residual feed intake (less efficient birds) [28], and reduction in feed efficiency is a negative consequence of HS [6]. In our experiment, HS led to higher relative abundance of genera such as *Bilophila*, *CHKCI001*, *GCA-900066575*, *Negativibacillus*, *Ruminococcaceae UCG-014*, *Christensenellaceae R-7 group*, *Tyzzerella*, and *[Eubacterium] hallii group* in the IL-M as well. There is not much known about the function and dynamic of these bacteria especially in the IL, but enrichment of these genera in birds exposed to HS might be an indicator of disrupted GIT health and integrity, that allows the overgrowth of such bacteria, and their contribution to the negative effects of HS warrants further research. Disruption of gut integrity in L1995 and L2015 occurs during HS [3].

Modern broiler genetic lines have significantly higher feed intake compared to their ancestors [3], which might make the GIT environment more unstable due to the rapid flow of digesta. The slower the passage rate, the longer will be the digesta retention in the GIT, allowing more time for contact between digestive enzymes and substrates as well as products of digestion and intestinal mucosa [29]. Thus, GIT microbial composition of modern broilers might differ from their ancestors. There was no effect of genetics on the diversity of microbial community, while composition of IL microbiota was affected by genetic lines. Higher abundance of *Faecalibacterium* is correlated with improved efficiency [30, 31], which is not the case in this experiment as we showed higher abundance of *Faecalibacterium* in ACRB and JF (low efficient) compared to L1995 and L2015 (high efficient) in the IL-M. Furthermore, relative abundance of Firmicutes was higher in the IL-M of L2015 compared to both JF and ACRB, and L1995 compared to JF; while in the IL-M relative abundance of *Lactobacillus* was higher in L1995 and L2015 compared to both JF and ACRB. This is correlated with better performance and FCR in L1995 and L2015 compared to ACRB and JF shown in a previous publication [3]. Comparison of GIT microbiota of hens with diverse feed efficiency showed higher abundance of *Lactobacillus* and lower abundance of *Faecalibacterium* in more efficient birds compared to their less efficient counterparts [13]. *Lactobacillus* is among the predominant bacterial genera in the GIT of broiler chickens [12, 32]. These bacteria have various beneficial effects which include immunomodulation, antagonistic activity against pathogens by lowering pH, production of bacteriostatic and bactericidal substances, competitive exclusion, enhancing mucosal barrier function by regulating tight junctions, and providing substrates (lactic acid) for butyrate producing bacteria [33–39]. This study supports previous studies that link higher *Lactobacillus* and lower *Faecalibacterium* abundance to better performance and FCR [13] and is in contrast with the studies which correlate lower *Lactobacillus* [23, 40] and higher *Faecalibacterium* [30, 31] abundance to performance. *Lactobacillus* is a genus with multiple species; thus, these differences might be due to the differences in abundance of various *Lactobacillus* species in experiments. Interestingly, compared to L1995 and L2015, relative abundance of *CHKCI001*, *Lactococcus*, *Staphylococcus*, *Weisella*, *Brachybacterium*, *Candidatus Arthromitus*, and *Rothia* were higher in the IL-L of JF; while relative abundance of *Ruminococcacea UCG-014*, *Mailhella*, and *Negativibacillus* were higher in the IL-M of JF. This indicates that the shift in microbial composition of IL-L and IL-M due to genetic selection is site specific. Another interesting finding was the higher relative abundance of *Butyricicoccus*, *Bacteroides*, *Subdoligranulum*, and *Mailhella* in IL-M of JF, ACRB, or both compared to L1995 and L2015. Some bacteria such as *Clostridium*, *Butyricicoccus, Faecalibacterium*, and *Subdoligranulum* are short chain fatty acid producers [28, 41]. Although some short chain fatty acid producers like *Oscillibacter* and *Butyricicoccus* are positively correlated with feed efficiency, others such as *Subdoligranulum* are negatively associated with feed efficiency in chickens [28]. Therefore, it is very important to understand the exact role of each bacterial species as well as the role and importance of each specific bacteria within the microbial community, before making any conclusion about their positive or negative correlation with performance parameters.

GIT microbiota cross feed each other and can provide metabolites for host nutrition as well [42]. Microbial metabolism increases energy yield and storage from the diet, regulates fat storage, and generates essential vitamins [43]. In broiler chickens economic traits such as growth rate and muscle development are directly correlated to appetite [44]. Thus, higher feed intake in modern broiler chickens might have reduced the dependency of host on microbial metabolites as a nutrient source. Enrichment of functional genes involved in amino acid metabolism, and metabolism of cofactors and vitamins in JF and ACRB, compared L2015, might be due to lower feed intake in these birds and the need for microbiota to synthesize these essential nutrients. Furthermore, due to the genetic selection, feed retention time in the GIT of modern broiler chickens is shorter [45]. The short retention time allows for a high feed intake despite the limitations to volume of the digestive system [46]. However, slow passage rate allows for longer digesta retention in the GIT, therefore contact time between digestive enzymes and substrates as well as products of digestion and intestinal mucosa will increase [29]. Thus, longer feed retention time in the GIT of JF and ACRB might favor more stable environment for the microbial community and allow microbiota to utilize the digesta more efficiently to produce various metabolites [35]. On the other hand, enrichment of functional genes involved in carbohydrate metabolism and lipid metabolism in the GIT of L2015 compared to JF and ACRB might be due to the higher availability of carbohydrates, because of higher feed intake [44]. Commercial broiler chickens’ diet is loaded with carbohydrates, which along with higher feed intake, might have led to the selection (cohabitation) of microbial community harboring enriched functional genes involved in carbohydrate metabolism in L2015 (including glycolysis/gluconeogenesis, pyruvate metabolism, and propanoate metabolism, and fructose and mannose metabolism). Short chain fatty acids (including propanoate) induce glucagon like peptide 1 secretion in the intestinal epithelial cells of chickens, which indicates the ability of the GIT microbiota to regulate hepatic lipogenesis [47].

Besides regulating host metabolism, GIT microbiota has a holistic role in the development of immune responses [42], and findings evidence the negative consequence of long-term selection for feed efficiency and body weight on the immune responses in chickens [1]. Enriched functional genes involved in immune system and endocrine system in JF and ACRB microbiota, compared to L2015, might indicate the impact of GIT microbiome on better immune responses in these lines.

To recap, this experiment highlighted the importance of environmental conditions and genetic composition on GIT microbial diversity, composition and function and provides invaluable data for developing individualized nutritional approaches for better health and alleviating the negative effects of chronic environmental stressors. As an instance, due to the shifts in microbial composition in HS compared to TN condition (especially taxa such as Proteobacteria, *Lactobacillus*, *Faecalibacterium*, *Ruminiclostridium 5* and *Blautia*), precision feeding of natural additives or probiotic species to maintain the levels of these bacteria during the HS should be considered in further research. Furthermore, some differences in microbial shifts in IL-L compared to IL-M due to the HS, emphasizes the importance of the target site when supplementing natural additives to the diet.

## Conclusion

HS affects community composition and function of ileal microbiota. The effect of HS on microbial composition of the ileum is site-specific and is more prominent on the ileal mucosa than ileal luminal content. Furthermore, taxonomic, and functional analysis showed a signature like microbiota for each genetic line. Shift in community function was largest in L2015 compared to JF, with no difference between L2015 and L1995. This highlights the effect of temperature and genetic composition on the diversity of ileal microbiota and function.

## Supplementary Information

**Additional file 1: Table S1.** Comparison of microbial community richness (Chao 1) in the luminal population. **Table S2.** Beta-diversity in luminal population. **Table S3.** Comparison of microbial community diversity in mucosal population (Simpson’s diversity). **Table S4.** Beta-diversity in mucosal population. **Figure S1.** Comparison in predicted microbiota function mucosal population between modern random bred (L2015) and jungle fowl (JF). **Figure S2.** Comparison in predicted microbiota function in mucosal population between modern random bred (L2015) and Athens-Canadian Random bred (ACRB).

## Data Availability

The 16S rRNA gene sequences determined in this study were deposited in the NCBI Sequence Read Archive (SRA) database (http://nih.gov/bioproject/browse; SRA accession # PRJNA794190).

## Abbreviations

ACRB: Athens-Canadian Random Bred
ASV: Amplicon sequence variant
d: Day
FDR: False discovery rate
GIT: Gastrointestinal tract
HS: Heat stress
IL: Ileum
IL-L: Ileal luminal bacterial population
IL-M: Ileal mucosal bacterial population
JF: Giant Jungle Fowl
KEGG: Kyoto encyclopedia of genes and genomes
L: Luminal
LAR: Low abundance reads
L1995: 1995 Random bred
L2015: Modern random bred
M: Mucosal
PCoA: Principal coordinate analysis
PERMANOVA: Non-parametric multivariate ANOVA
PICRUSt: Phylogenetic Investigation of Communities by Reconstruction of Unobserved States
QIIME: Quantitative Insight Into Microbial Ecology
SRA: NCBI sequence read archive
STAMP: Statystical analysis of metagenomic profiles
TN: Thermoneutral
