# Supplementary material for: Ileal microbial composition in genetically distinct chicken lines reared under normal or high ambient temperatures
Source: Anim Microbiome. 2022 Apr 21;4:28. doi: 10.1186/s42523-022-00183-y (PMC9028080; doi:10.1186/s42523-022-00183-y)

**Additional file 1**

**Table S1**. Comparison of microbial community richness (Chao1 diversity index) in the ileal content of birds from 4 genetic lines using Pairwise Kruskal-Wallis test (d 56).^1^

| **Group 1** | **Group 2** | **H** | **p-value** | **q-value** |
| --- | --- | --- | --- | --- |
| **ACRB** | JF | 0.01 | 0.908 | 0.908 |
| **ACRB** | L1995 | 0.36 | 0.544 | 0.653 |
| **ACRB** | L2015 | 1.33 | 0.248 | 0.490 |
| **JF** | L1995 | 0.96 | 0.326 | 0.490 |
| **JF** | L2015 | 3.85 | 0.050 | 0.298 |
| **L1995** | L2015 | 1.02 | 0.312 | 0.490 |

| **Group 1** | **Group 2** | **H** | **p-value** | **q-value** |
| --- | --- | --- | --- | --- |
| **HS** | TN | 12.43 | <0.001 | <0.001 |

^1^ Treatments include: Giant Jungle Fowl (JF), Athens Canadian Random Bred (ACRB), 1995 Random Bred (L1995), and Modern Random Bred (L2015) lines raised under thermoneutral (TN, d 29-56; 24°C) or subjected to chronic cyclic heat stress (HS; d 29-56; 36°C for 8 h/d) conditions.

**Table S2.** Group significance and pairwise PERMANOVA statistics based on unweighted UniFrac distance matrix on ileal content samples on d 56.^1^

| **Number of groups** | **Test statistic** | **Sample size** | **p-value^2^** |
| --- | --- | --- | --- |
| **8** | 1.94 | 48 | 0.001 |

| **Group 1** | **Group 2** | **Sample size** | **pseudo-F** | **p-value^2^** | **q-value** |
| --- | --- | --- | --- | --- | --- |
| **L1995-HS** | L1995-TN | 12 | 1.09 | 0.353 | 0.395 |
| **L1995-HS** | L2015-HS | 12 | 0.66 | 0.811 | 0.811 |
| **L1995-HS** | L2015-TN | 12 | 1.58 | 0.091 | 0.149 |
| **L1995-HS** | ACRB-HS | 12 | 2.79 | 0.009 | 0.036 |
| **L1995-HS** | ACRB-TN | 12 | 1.28 | 0.187 | 0.238 |
| **L1995-HS** | JF-HS | 12 | 1.59 | 0.055 | 0.110 |
| **L1995-HS** | JF-TN | 12 | 2.15 | 0.003 | 0.028 |
| **L1995-TN** | L2015-HS | 12 | 1.12 | 0.347 | 0.395 |
| **L1995-TN** | L2015-TN | 12 | 0.88 | 0.561 | 0.581 |
| **L1995-TN** | ACRB-HS | 12 | 4.12 | 0.007 | 0.032 |
| **L1995-TN** | ACRB-TN | 12 | 1.43 | 0.131 | 0.193 |
| **L1995-TN** | JF-HS | 12 | 1.85 | 0.068 | 0.119 |
| **L1995-TN** | JF-TN | 12 | 3.12 | 0.011 | 0.038 |
| **L2015-HS** | L2015-TN | 12 | 1.31 | 0.199 | 0.242 |
| **L2015-HS** | ACRB-HS | 12 | 3.48 | 0.006 | 0.032 |
| **L2015-HS** | ACRB-TN | 12 | 1.82 | 0.016 | 0.044 |
| **L2015-HS** | JF-HS | 12 | 1.89 | 0.026 | 0.066 |
| **L2015-HS** | JF-TN | 12 | 2.81 | 0.003 | 0.028 |
| **L2015-TN** | ACRB-HS | 12 | 4.51 | 0.005 | 0.032 |
| **L2015-TN** | ACRB-TN | 12 | 1.70 | 0.032 | 0.068 |
| **L2015-TN** | JF-HS | 12 | 1.97 | 0.031 | 0.068 |
| **L2015-TN** | JF-TN | 12 | 4.03 | 0.002 | 0.028 |
| **ACRB-HS** | ACRB-TN | 12 | 1.44 | 0.114 | 0.177 |
| **ACRB-HS** | JF-HS | 12 | 1.23 | 0.164 | 0.218 |
| **ACRB-HS** | JF-TN | 12 | 2.44 | 0.016 | 0.044 |
| **ACRB-TN** | JF-HS | 12 | 1.00 | 0.428 | 0.460 |
| **ACRB-TN** | JF-TN | 12 | 1.39 | 0.14 | 0.196 |
| **JF-HS** | JF-TN | 12 | 1.35 | 0.068 | 0.119 |

^1^ Treatments include: Giant Jungle Fowl (JF), Athens Canadian Random Bred (ACRB), 1995 Random Bred (L1995), and Modern Random Bred (L2015) lines raised under thermoneutral (TN, d 29-56; 24°C) or subjected to chronic cyclic heat stress (HS; d 29-56; 36°C for 8 h/d) conditions.

^2^ *P*-values were calculated based on 999 permutation tests.

**Table S3**. Comparison of microbial community richness and evenness (Simpson’s diversity index) in the ileal mucosal scrapings of birds from 4 genetic lines using Pairwise Kruskal-Wallis test (d 56).^1^

| **Group 1** | **Group 2** | **H** | **p-value** | **q-value** |
| --- | --- | --- | --- | --- |
| **ACRB** | JF | 2.43 | 0.119 | 0.267 |
| **ACRB** | L1995 | 2.25 | 0.133 | 0.267 |
| **ACRB** | L2015 | 3.41 | 0.065 | 0.267 |
| **JF** | L1995 | 0.00 | 0.954 | 0.954 |
| **JF** | L2015 | 0.00 | 0.954 | 0.954 |
| **L1995** | L2015 | 0.03 | 0.862 | 0.954 |

| **Group 1** | **Group 2** | **H** | **p-value** | **q-value** |
| --- | --- | --- | --- | --- |
| **HS** | TN | 8.57 | 0.003 | 0.003 |

^1^ Treatments include: Giant Jungle Fowl (JF), Athens Canadian Random Bred (ACRB), 1995 Random Bred (L1995), and Modern Random Bred (L2015) lines raised under thermoneutral (TN, d 29-56; 24°C) or subjected to chronic cyclic heat stress (HS; d 29-56; 36°C for 8 h/d) conditions.

**Table S4.** Group and pairwise PERMANOVA statistics based on unweighted UniFrac distance matrix on ileal mucosal scraping samples on d 56.^1^

| **Number of groups** | **Test statistic** | **Sample size** | **p-value^2^** |
| --- | --- | --- | --- |
| **8** | 1.170 | 47 | 0.041 |

| **Group 1** | **Group 2** | **Sample size** | **pseudo-F** | **p-value^2^** | **q-value** |
| --- | --- | --- | --- | --- | --- |
| **ACRB-HS** | ACRB-TN | 12 | 1.16 | 0.157 | 0.366 |
| **ACRB-HS** | JF-HS | 12 | 0.97 | 0.434 | 0.578 |
| **ACRB-HS** | JF-TN | 12 | 1.13 | 0.224 | 0.418 |
| **ACRB-HS** | L1995-HS | 12 | 0.83 | 0.797 | 0.807 |
| **ACRB-HS** | L1995-TN | 11 | 1.08 | 0.280 | 0.446 |
| **ACRB-HS** | L2015-HS | 12 | 0.90 | 0.605 | 0.678 |
| **ACRB-HS** | L2015-TN | 12 | 1.07 | 0.284 | 0.446 |
| **ACRB-TN** | JF-HS | 12 | 1.81 | 0.001 | 0.003 |
| **ACRB-TN** | JF-TN | 12 | 1.62 | 0.032 | 0.205 |
| **ACRB-TN** | L1995-HS | 12 | 1.26 | 0.022 | 0.205 |
| **ACRB-TN** | L1995-TN | 11 | 1.43 | 0.089 | 0.266 |
| **ACRB-TN** | L2015-HS | 12 | 1.54 | 0.009 | 0.126 |
| **ACRB-TN** | L2015-TN | 12 | 1.37 | 0.060 | 0.240 |
| **JF-HS** | JF-TN | 12 | 0.86 | 0.776 | 0.807 |
| **JF-HS** | L1995-HS | 12 | 1.45 | 0.037 | 0.205 |
| **JF-HS** | L1995-TN | 11 | 0.83 | 0.807 | 0.807 |
| **JF-HS** | L2015-HS | 12 | 0.93 | 0.577 | 0.678 |
| **JF-HS** | L2015-TN | 12 | 1.08 | 0.287 | 0.446 |
| **JF-TN** | L1995-HS | 12 | 1.40 | 0.044 | 0.205 |
| **JF-TN** | L1995-TN | 11 | 0.90 | 0.606 | 0.678 |
| **JF-TN** | L2015-HS | 12 | 1.15 | 0.180 | 0.368 |
| **JF-TN** | L2015-TN | 12 | 1.28 | 0.114 | 0.290 |
| **L1995-HS** | L1995-TN | 11 | 1.34 | 0.095 | 0.266 |
| **L1995-HS** | L2015-HS | 12 | 1.27 | 0.075 | 0.262 |
| **L1995-HS** | L2015-TN | 12 | 1.18 | 0.184 | 0.368 |
| **L1995-TN** | L2015-HS | 11 | 1.00 | 0.400 | 0.560 |
| **L1995-TN** | L2015-TN | 11 | 0.87 | 0.571 | 0.678 |
| **L2015-HS** | L2015-TN | 12 | 1.02 | 0.381 | 0.560 |

^1^ Treatments include: Giant Jungle Fowl (JF), Athens Canadian Random Bred (ACRB), 1995 Random Bred (L1995), and Modern Random Bred (L2015) lines raised under thermoneutral (TN, d 29-56; 24°C) or subjected to chronic cyclic heat stress (HS; d 29-56; 36°C for 8 h/d) conditions.

^2^ *P*-values were calculated based on 999 permutation tests.

**Figure S1**. Predicted functions of ileal mucosal scrapings microbiota in modern random bred chickens (L2015) compared to their ancestor jungle fowl (JF) on d 56. Differentially regulated metabolic pathways are shown. (n = 12/genetic line)


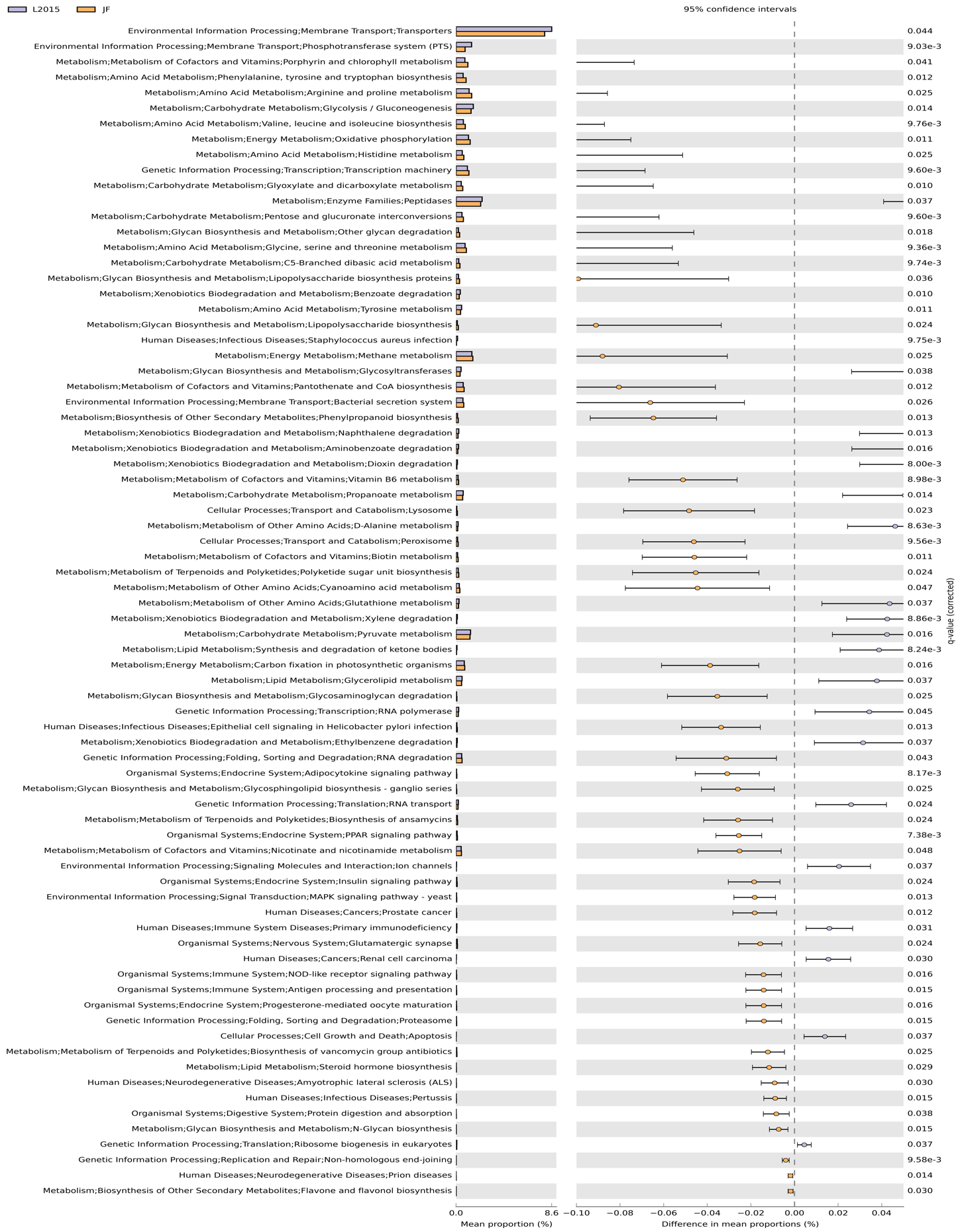


**Figure S2**. Predicted functions of ileal mucosal scrapings microbiota in modern random bred chickens (L2015) compared to Athens-Canadian Random bred chickens (ACRB) on d 56 Differentially regulated metabolic pathways are shown. (n = 12/genetic line).


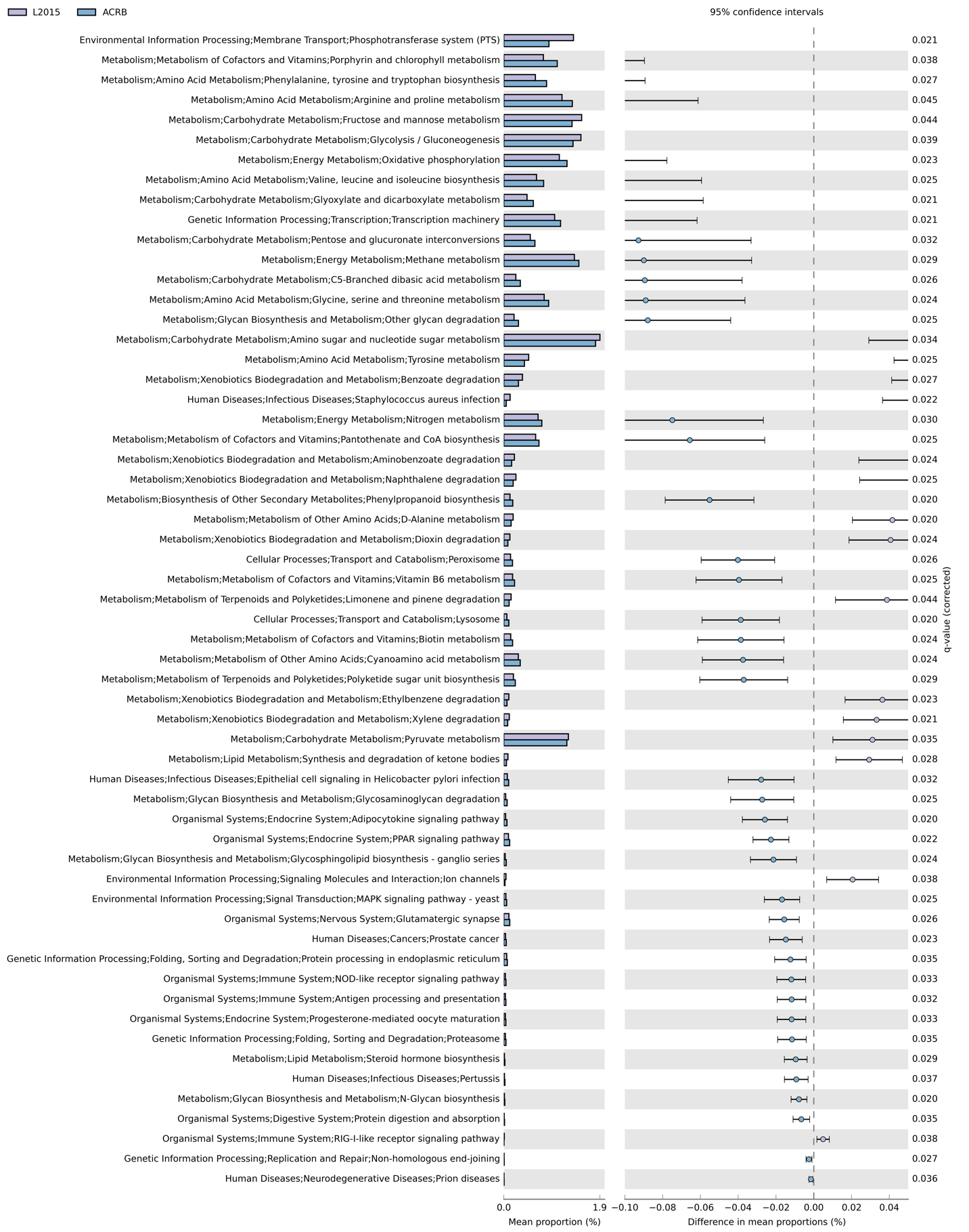

Supplement: Supplementary file 1 — Additional file 1: Table S1. Comparison of microbial community richness (Chao 1) in the luminal population. Table S2. Beta-diversity in luminal population. Table S3. Comparison of microbial community diversity in mucosal population (Simpson’s diversity). Table S4. Beta-diversity in mucosal population. Figure S1. Comparison in predicted microbiota function mucosal population between modern random bred (L2015) and jungle fowl (JF). Figure S2. Comparison in predicted microbiota function in mucosal population between modern random bred (L2015) and Athens-Canadian Random bred (ACRB). [file 42523_2022_183_MOESM1_ESM.docx]
